# Cost-Effective Fish Volume Estimation in Aquaculture Using Infrared Imaging and Multi-Modal Deep Learning

**DOI:** 10.3390/s26041221

**Published:** 2026-02-13

**Authors:** Like Zhang, Yanling Han, Ge Song, Jing Wang, Ping Ma

**Affiliations:** College of Information Technology, Shanghai Ocean University, Shanghai 201306, China

**Keywords:** aquaculture, fish volume estimation, infrared imaging, cost-effective, multi-modal deep learning, biomass monitoring, synthetic data generation

## Abstract

Accurate fish volume estimation is essential for sustainable aquaculture management, yet traditional methods are invasive and costly, while existing non-invasive approaches rely on expensive multi-sensor setups. This study proposes a cost-effective infrared (IR)-only pipeline that reconstructs depth and Red Green Blue (RGB) from low-cost infrared videos (<USD 100 per camera), enabling scalable biomass monitoring in dense tanks. The pipeline integrates five modules: IR-to-depth estimation with contour-guided attention and smoothing loss; IR-to-RGB generation via texture-conditioned injection and water-adaptive loss; detection and tracking using cross-modal fusion and behavior-constrained Kalman filtering; instance segmentation with depth-guided branches and deformation-adaptive loss; and volume estimation through trajectory–depth Transformer fusion with refraction correction. Trained on a curated dataset of 166 goldfish across 124 videos (8–16 fish/tank), the system achieves Mean Absolute Error (MAE) of 0.85 cm^3^ and coefficient of determination (R^2^) of 0.961 for volume estimation, outperforming state-of-the-art methods by 19–41% while reducing hardware costs by 80%. This work advances precision aquaculture by providing robust, deployable tools for feed optimization and health monitoring, promoting environmental sustainability amid rising global seafood demand.

## 1. Introduction

**Figure 1:**
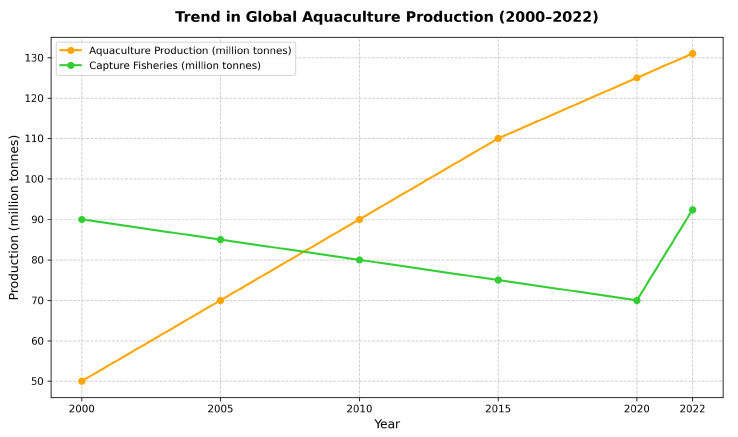
Trend in global aquaculture production (2000–2022). Data source: FAO (2023).

Aquaculture, the controlled cultivation of aquatic organisms such as fish, shellfish [1], and algae, has emerged as a critical component of global food security and economic development [2,3]. According to the Food and Agriculture Organization (FAO) of the United Nations, global fisheries and aquaculture production reached a record level in recent years [4]. As illustrated in Figure 1, aquaculture contributed approximately 130.9 million tons in 2022 [5], surpassing capture fisheries for the first time in history. This trend—driven by expanding demand for protein-rich seafood amid a growing world population projected to reach 9.7 billion by 2050—highlights both the sector’s importance and the sustainability challenges it faces [6,7]. In particular, finfish production dominates, accounting for over 90 million tons annually, with species like tilapia, carp, and salmon being staples in both freshwater and marine systems. The sector supports millions of livelihoods and generates substantial economic value; for example, total trade in aquatic products exceeded USD 472 billion in 2022 [8,9]. However, as aquaculture intensifies to meet rising demand—projected to grow further by 2033—the need for sustainable practices becomes paramount to mitigate environmental impacts such as habitat degradation, disease outbreaks, and pollution from overfeeding.

Central to sustainable aquaculture management is the accurate monitoring of fish growth and health, particularly through biomass estimation, which informs feeding strategies, stocking densities, and harvest timing. Biomass—often approximated via volume or weight measurements—directly influences feed conversion ratios (FCRs) [10]. Inefficient monitoring can lead to overfeeding (wasting a substantial fraction of feed) or underfeeding (stunting growth and increasing mortality). Traditional methods for biomass assessment, such as manual netting and weighing, are invasive, labor-intensive, and stressful for fish, potentially causing injuries, disease transmission, or altered behaviors that bias measurements [11]. These approaches are particularly challenging in large-scale operations, where frequent sampling disrupts production and incurs significant costs. Moreover, in dense farming environments (e.g., 8–16 fish per cubic meter in some intensive setups), occlusions and rapid movements further exacerbate measurement errors.

**Figure 2:**
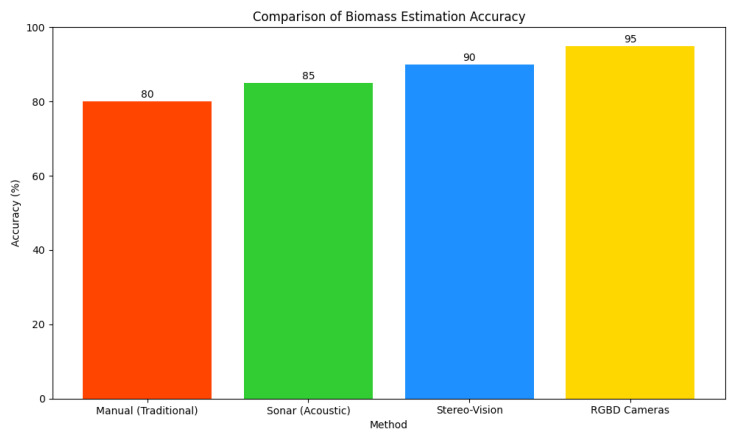
Comparison of biomass estimation accuracy across traditional (manual) and modern (sensor-based) methods.

Non-invasive alternatives have gained traction, leveraging sensors and imaging technologies to reduce disturbance (see comparison in Figure 2). Acoustic methods (e.g., sonar) can approximate bulk biomass but have low resolution in turbid waters and cannot distinguish individuals within schools. Optical approaches, such as stereo vision and Red Green Blue Depth (RGB-D) cameras, enable 3D reconstruction for length and volume estimation but require clear visibility and are sensitive to turbidity, lighting variations, and biofouling [12]. RGB-D systems also tend to be more expensive and computationally demanding, which limits scalability in resource-constrained farms. Recent advances in computer vision and deep learning have improved fish counting, tracking, and morphological analysis, using detectors and segmentation networks adapted for underwater conditions. Image enhancement (Generative Adversarial Networks [GANs], diffusion models) and multi-modal fusion (RGB, infrared, acoustic) help address low-visibility issues [13]; however, many existing systems still rely on multi-sensor setups and substantial compute resources, restricting adoption.

The rationale for employing a multi-modal deep learning framework primarily stems from the inherent limitations of single-channel infrared imaging. While IR sensors are cost-effective and illumination-invariant, they lack the chromatic texture cues required for robust feature matching in tracking algorithms and the geometric depth information essential for volumetric quantification. By synthesizing pseudo-RGB and depth modalities from IR inputs, our approach recovers these missing dimensions, leveraging the rich feature representations of pre-trained networks (typically trained on RGB data) while maintaining the low hardware cost of IR sensors. To address these limitations, this study proposes a cost-effective pipeline that uses low-cost infrared (IR) cameras as the primary sensor and reconstructs depth and RGB cues from IR input via learned models. By training on RGB-D datasets and deploying on inexpensive IR-only hardware (camera cost < USD 100 per unit), the system reduces hardware expense while enabling scalable monitoring. The pipeline comprises five modules: (1) an IR-to-depth estimator with contour-guided attention; (2) an IR-to-RGB generative model; (3) a cross-modal detection and tracking module; (4) a depth-guided instance segmentation network; (5) a trajectory–depth fusion module for physics-aware volume estimation.

We evaluate our approach on a purpose-built dataset collected in controlled tank experiments, consisting of recordings from 166 fish across 124 ten-minute videos with varying densities. Annotations include instance masks and bounding boxes; videos are segmented into short clips (3 s) aligned to typical swimming speeds to support temporal modeling. We report Mean Absolute Error (MAE) for volume estimation and Intersection over Union (IoU) for segmentation as primary metrics [14].

The contributions of this work are threefold: (1) a practical IR-only pipeline for individual fish volume estimation that substantially lowers sensor cost while maintaining accuracy; (2) several aquaculture-specific algorithmic innovations improving robustness to underwater artifacts; (3) a curated dataset and evaluation protocol for multi-fish volume monitoring. The remainder of this paper is organized as follows: Section 2 reviews related work; Section 3 describes the proposed methodology; Section 4 presents experiments and analysis; and Section 5 concludes with future directions [15].

## 2. Related Works

Fish volume and biomass estimation in aquaculture has attracted substantial research attention because of its direct role in feeding optimization, harvest scheduling, and resource management. Advances in computer vision and learning-based methods have enabled non-invasive monitoring at scales and speeds not possible with traditional netting and weighing. In what follows, we review representative literature in three topical areas: (1) fish detection and segmentation in underwater and low-visibility environments; (2) fish size and biomass estimation (including stereo- and single-camera approaches); (3) multi-modal and generative approaches that address missing depth or color information in low-cost setups.

### 2.1. Fish Detection and Segmentation in Underwater and Low-Visibility Settings

A large body of work has focused on making detection and instance segmentation robust to underwater imaging challenges (turbidity, low contrast, motion blur, and occlusion). Surveys and reviews summarize these developments and benchmark datasets. Deep CNN detectors (YOLO-family, Faster/Mask R-CNN variants, and recent transformer-based detectors) [16] have been widely adopted and adapted for aquaculture contexts. For instance, recent works have introduced salient object detection-guided segmentation for fish phenotypes [17], improving accuracy in complex scenes. Another approach proposes an underwater image segmentation model tailored for aquaculture, handling turbidity and occlusions effectively through enhanced convolutional networks. Multi-task networks like FDMNet combine detection and segmentation for fish disease monitoring, achieving high precision in low-visibility conditions [18]. Self-supervised learning methods have been developed to overcome annotation bottlenecks, enabling robust segmentation in underwater videos with minimal labeled data. Dual-stream frameworks such as DeepFishNET+ enhance detection and classification by addressing image degradation in aquaculture environments. Improved deep learning frameworks for fish segmentation in underwater videos provide non-invasive, rapid procedures for detecting fish behavior in mixed polyculture systems. Comprehensive studies categorize fish monitoring applications into domains like detection, recognition, biomass estimation, behavior classification, and health analysis. Enhanced YOLOv5 models improve fish recognition and classification in complex backgrounds and low-light conditions [19]. Systematic literature reviews highlight AI methodologies in aquaculture, including improved models for underwater species recognition. Multi-feature fusion models for fish image segmentation in aquaculture environments address color cast, unbalanced contrast, and blur. Salient object detection guided by multi-task learning enables precise fish phenotype segmentation in high-density underwater scenes. Enhanced soft attention mechanisms improve segmentation accuracy in complex aquaculture environments [20]. Two-mode underwater smart sensors combine sonar and stereo cameras for precision aquaculture monitoring. For low-visibility sensing modalities such as imaging sonar or above-water infrared cameras, Mask R-CNN and related instance segmentation pipelines have also been successfully applied after appropriate preprocessing (e.g., feature-standardization or CRF-based normalization). Above-water IR deployments and comparisons of IR-based backbones for fish detection have demonstrated competitive AP values while offering robustness to lighting changes [21]. These detection/segmentation advances form the backbone for downstream sizing and biomass estimation. Moreover, recent studies have expanded computer vision applications to crustacean monitoring, such as the work by Morimoto et al. [22], which utilized image analysis for abnormal behavior detection in infected shrimp, demonstrating the versatility of visual monitoring in aquaculture health management.

### 2.2. Fish Size and Biomass Estimation

Traditional non-invasive size estimation commonly uses stereo vision or calibrated monocular rigs to obtain geometric measures (length, height) and compute weight/biomass via allometric relationships (length–weight models) [23]. Recent stereo-based, end-to-end systems combine segmentation/pose estimation with stereo depth reconstruction and regression to predict weight with low MAE [24]. When stereo or depth sensors are available, researchers have obtained very accurate length/weight predictions (e.g., sub-cm length errors and high R2 values) but at the cost of additional hardware and computational overhead. Single-image methods and learned regressors have also shown promising results for constrained viewpoints, using features like contour area, chord lengths, and learned deep features to estimate mass with MAPE/MAE in acceptable ranges for certain species. For example, multi-modal deep learning has been applied to shrimp biomass estimation, fusing visual and sensor data for improved accuracy in aquaculture. Frameworks addressing occluded features in fish biomass estimation combine imputation and regression to handle dense scenes. Two-stream transformers enhance weight estimation by integrating multi-modal data [25], reducing errors in non-contact measurements. Machine vision applications for welfare monitoring include size estimation techniques that leverage computer vision for precise biomass calculations in various aquaculture settings. Non-invasive fish biometrics validate computer vision methods for biomass estimation and development monitoring. FishKP-YOLOv11 provides a non-contact framework for estimating fish size and mass, addressing inaccuracies in dense environments. Comprehensive surveys review computer vision approaches for fish monitoring, including biomass estimation [26]. Fish biomass estimation under occluded features uses imputation and regression frameworks for accurate predictions. However, most prior work focuses on single-fish or sparse scenes under controlled viewpoints; multi-fish, highly occluded tank scenarios remain challenging.

### 2.3. Multi-Modal and Generative Approaches for Missing Depth/Color Cues

To reduce hardware cost while retaining useful geometric cues, a number of works exploit multi-modal fusion (visual + acoustic + environmental sensors) or generative mapping (e.g., RGB-depth or IR-RGB) to synthesize missing modalities. Multi-modal fusion architectures have been shown to improve robustness for tasks like feeding detection and behavior analysis. For depth/appearance synthesis, general monocular and self-supervised depth estimation techniques (e.g., Eigen et al., Monodepth2 [27]) provide strong priors and training strategies that can be adapted to underwater or IR domain. Recent aquaculture-specific efforts explore combining stereo-derived ground truth and learned monocular estimators to produce usable depth for biomass estimation while lowering sensor costs. Cross-modal complementarity learning enhances fish feeding intensity prediction by fusing visual and acoustic data in underwater environments. Multi-attention-based underwater depth estimation models like MAU-Depth offer lightweight self-supervised solutions for monocular setups in aquaculture [28]. Underwater monocular metric depth estimation benchmarks evaluate zero-shot and fine-tuned models on real-world datasets, addressing challenges like missing depth cues. Physics-informed knowledge transfer methods enable real-time depth estimation from monocular images, fusing with acoustic sensors for enhanced accuracy. Multi-modal large language models have been tuned for fish detection, handling missing modalities in monitoring systems [29]. Application of generative AI in aquaculture evaluates advantages and challenges in unique environments. Underwater fish image recognition using knowledge graphs and semantic enhancement frameworks improves degraded image processing. Multi-modal vision-based systems for fish environment and growth monitoring utilize NeuroVI and RGB images for novel segmentation approaches [30]. Multi-modal fusion image enhancement techniques with CFEC-YOLOv7 models excel in underwater object detection. Semi-supervised underwater image enhancement methods use multi-modal contrastive learning for improved quality [31]. FishAI 2.0 leverages multi-modal few-shot learning for marine fish image classification. Deep learning reviews in sustainable aquaculture cover opportunities in detection, counting, and growth prediction [32]. Nevertheless, explicit IR-to-depth or IR-to-RGB mapping tailored to multi-fish aquaculture video—together with physics-aware corrections (refraction, optical distortion) and deformation-aware segmentation losses—is still under-explored.

### 2.4. This Work in Context

Building on these lines of research, our pipeline targets the under-studied intersection: cost-sensitive, IR-only sensing for multi-fish volume estimation in dense tank environments. We leverage recent monocular-depth and generative model advances as priors, but introduce aquaculture-specific modules (contour-guided depth attention, texture-conditioned IR-to-RGB injection [33], deformation-adaptive segmentation losses, and trajectory–depth fusion with refraction correction) to handle multi-fish occlusions, thermal/IR noise, and deformation during swimming. This positions our contributions as a practical complement to high-accuracy stereo and multi-sensor solutions while addressing real deployment constraints faced in many farms.

## 3. Materials and Methods

This section provides a detailed overview of the datasets, experimental setup, and methodological framework adopted in our study. The proposed system is designed as a modular yet end-to-end pipeline that converts low-cost infrared (IR) video sequences into accurate volumetric fish estimates. Its architecture emphasizes scalability and real-time applicability in aquaculture environments, where challenges such as IR noise, underwater refraction, occlusion, and fish deformation frequently occur. Each module is purposefully constructed to build upon the preceding one, enabling independent optimization while maintaining a differentiable structure that supports joint fine-tuning during training. The following subsections outline the overall system pipeline and describe each key module in detail.

### 3.1. Dataset Collection and Preparation

A robust and representative dataset is foundational to training and validating our pipeline, ensuring its generalization to diverse ornamental aquaculture scenarios. To this end, we conducted controlled experiments in a laboratory setting mimicking commercial ornamental fish tanks. The setup featured a transparent acrylic tank measuring 1m×1m×0.5m, filled with dechlorinated freshwater maintained at a pH of 7.0–7.5 and temperature of 20–25 °C, conditions optimal for goldfish (*Carassius auratus*), the primary species used in this study due to its prominence in the global ornamental fish trade.

We sourced 166 healthy juvenile goldfish from a local hatchery, with individual lengths ranging from 5 to 15 cm and weights from 10 to 200 g, representing typical sizes monitored in ornamental aquaculture. To simulate varying stocking densities—a critical factor in ornamental tanks where overcrowding can affect fish health and aesthetic appeal—we distributed the fish into groups of 8, 9, 10, 11, 12, 13, 14, 15, and 16 individuals per tank. This range (8–16 fish/m^3^) aligns with standard practices in goldfish aquariums, capturing realistic interactions like schooling, fin displays, and partial occlusions that challenge computer vision systems.

**Figure 3:**
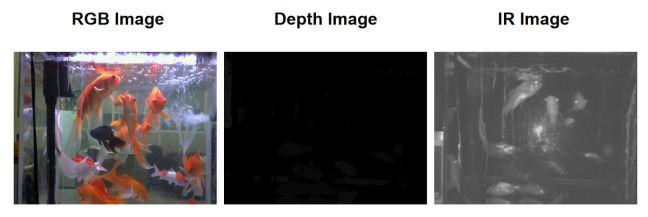
Sample frames from the three dataset modalities (RGB, Infrared, Depth).

Data acquisition employed an Intel RealSense D435 RGBD camera (Intel, Santa Clara, CA, USA) mounted 1 m above the tank surface, oriented downward for top-view coverage. Videos were recorded at 30 frames per second (FPS) with a resolution of 1280×720 pixels, resulting in 124 videos, each lasting 10 min (18,000 frames per video). The multi-modal streams included the following (Figure 3):

- Infrared (IR) channel: Capturing thermal signatures, resilient to lighting variations and water clarity typical in ornamental setups.
- RGB channel: Providing color and texture information, critical for capturing goldfish’s vibrant patterns for generative model supervision.
- Depth channel: Offering metric depth maps (0.5–2 m range) as ground truth for 3D reconstruction.

To incorporate environmental variability, recordings were performed under mixed lighting conditions (natural daylight and artificial LED lights at 500–1000 lux), with occasional water agitation to simulate ripples and bubbles common in decorative tanks. Behavioral diversity was encouraged through timed feeding sessions and gentle water currents (0.1–0.3 m/s flow rate), eliciting natural swimming patterns, including erratic fin movements and schooling displays unique to goldfish.

To ensure the dataset’s representativeness and robustness, we meticulously designed its composition to reflect the morphological, behavioral, and environmental diversity of goldfish in ornamental aquaculture. Table 1 details the distribution of goldfish varieties, age/size categories, and health/gender variations. We ensured a balanced representation of common (30%), fantail (30%), lionhead (20%), and comet (20%) varieties, alongside an equal split between juvenile and adult fish (50% each). This diversity is critical for training a model capable of handling the varied fin shapes and body sizes encountered in real-world settings.

Table 2 further outlines the density and interaction distribution, with densities ranging from low (30%) to high (30%) and a medium range (40%), alongside behavior types such as schooling (40%) and fin display (10%), which are essential for addressing occlusion and motion challenges. Environmental variations, as shown in Table 3, include lighting conditions (40% natural, 40% artificial LED, 20% mixed) and water quality states (60% clear, 20% mild turbidity, 20% decorated), ensuring the model generalizes across typical tank conditions.

The annotation and processing summary in Table 4 quantifies the dataset, comprising 124 videos (approx. 2.2 million raw frames), with 40,000 annotated keyframes and 160,000 clips. Augmentation strategies (2× geometric, 2× intensity, 3× domain-specific) were applied to enhance robustness. The dataset was split into 70% training, 15% validation, and 15% testing sets, stratified by these factors to mitigate bias.

**Figure 4:**
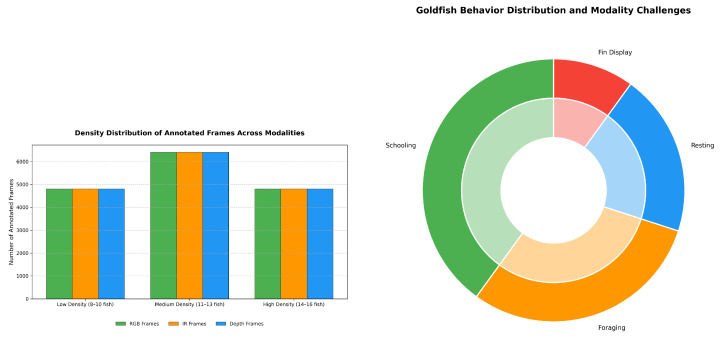
Distribution of density (**left**) and behavior subcategories (**right**) in the dataset.

**Figure 5:**
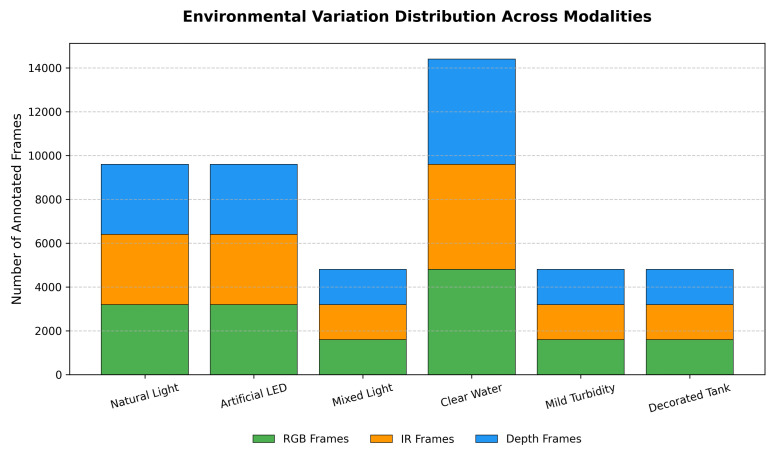
Distribution of environmental variations across modalities in the dataset.

To visually analyze the dataset’s composition, we present key distributions in Figure 4 and Figure 5. Figure 4 illustrates the density distribution (left) and behavior subcategories (right), highlighting a balanced representation across low (30%), medium (40%), and high (30%) densities, with schooling (40%) and foraging (30%) dominating behaviors. This balance is crucial for training the detection and tracking modules to handle occlusions and dynamic movements. Figure 5 depicts the environmental variation distribution, showing a significant portion under clear water (60%) and natural/artificial lighting (40% each), which supports the model’s robustness against common tank conditions. These visualizations underscore the dataset’s comprehensive coverage, enabling the pipeline to generalize across diverse scenarios.

Annotation was a labor-intensive but crucial step to generate high-fidelity labels. Bounding boxes were drawn using LabelImg (version 1.8.6), specifying fish locations and IDs for multi-object tracking. Instance segmentation masks were created with LabelMe (version 5.0.1), precisely outlining goldfish contours while accounting for pronounced fin movements and body flexions. Given the observed average swimming speeds of 0.1–0.4 m/s (quantified from preliminary motion analysis), we temporally segmented videos into 3 s clips (90 frames each). This clip length balances efficient processing with capturing meaningful trajectories and deformations, reducing the risk of tracking drift in longer sequences [34].

For ground truth volumes, post-recording, representative goldfish from each density group were gently removed and scanned using a high-precision EinScan Pro 2X 3D scanner (Shining 3D, Hangzhou, China), yielding volumetric measurements in cm^3^ with an accuracy of ±0.05 mm, suitable for the smaller scale of goldfish (10–1000 cm^3^). These were cross-verified with the Archimedes’ principle (water displacement) for consistency, achieving errors below 2%. The dataset thus encompasses not only 2D annotations but also 3D references, enabling supervised training across modules.

To augment the dataset and enhance model robustness, we applied a suite of transformations:

- Geometric: Random rotations (±15°), horizontal/vertical flips, and affine shearing (up to 10°).
- Intensity: Brightness/contrast adjustments (±20%), histogram equalization for IR normalization.
- Domain-specific: Synthetic water noise via Gaussian blurring (σ=0.01–0.05) and ripple simulations using sinusoidal wave functions:
  (1) $$I^{\prime }\left(x,y\right)=I\left(x+A\sin\left(2\pi fy+\phi \right),y+A\sin\left(2\pi fx+\phi \right)\right),$$
  where A=5 pixels (amplitude), f=0.05 (frequency), and ϕ∈[0,2π] (phase).

**Figure 6:**
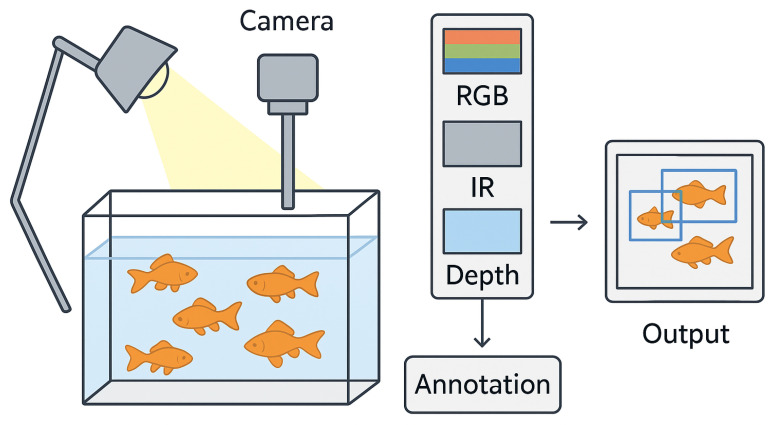
The workflow of dataset preparation and processing.

The augmented dataset was split into training (70%), validation (15%), and testing (15%) sets, stratified by fish density, behavior type (e.g., schooling vs. isolated), and environmental conditions to mitigate bias. The overall workflow of dataset preparation and processing is illustrated in Figure 6. This preparation ensures that the pipeline’s inputs are diverse and realistic, directly supporting the IR-centric inference paradigm that minimizes hardware costs in ornamental aquaculture deployment.

### 3.2. Overall Pipeline

**Figure 7:**
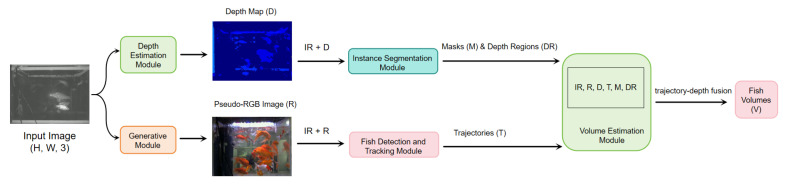
Overall framework of our proposed model.

Figure 7 illustrates the proposed end-to-end framework. Unlike traditional cascaded systems where errors propagate independently between isolated modules, our architecture fosters synergistic feature learning. The transition from raw IR input to volumetric output involves explicit intermediate representations (Depth and Pseudo-RGB) that effectively bridge the domain gap. This design contrasts with “black-box” direct regression models by enforcing physical constraints (e.g., depth consistency) and enhancing interpretability. The differentiable connectivity ensures that gradient signals from the volume loss can fine-tune upstream feature extractors, optimizing the entire pipeline for the specific challenges of underwater occlusion and refraction.

The proposed pipeline processes raw IR video inputs through a sequence of modules, progressively enhancing information from 2D thermal intensity to 3D fish volume representation. The framework is trained with full RGBD supervision but relies solely on single-channel IR inputs during inference, reducing hardware cost by approximately 80% compared to RGBD systems (USD 100 vs. USD 500 per unit). This makes it well suited for large-scale, cost-efficient deployment in aquaculture monitoring systems.

Given an IR video clip $I_{\mathrm{IR}}\in R^{T\times H\times W\times 1}$ (T=90 frames for 3 s at 30 FPS), the pipeline computes depth map D and pseudo-RGB image R:

(2) $$D=f_{\mathrm{depth}}\left(I_{\mathrm{IR}}\right),\;R=f_{\mathrm{gen}}\left(I_{\mathrm{IR}}\right).$$

Subsequently, the tracking module generates trajectories T:

(3) $$T=f_{\mathrm{track}}\left(I_{\mathrm{IR}},R\right)=\left\{\left(b_{i}^{t},{\mathrm{id}}_{i}\right)\;|\;i=1\ldots N,\;t=1\ldots T\right\},$$

where $b_{i}^{t}={\left[x,y,w,h\right]}^{T}$ denotes the bounding box of target *i* at frame *t*. The segmentation module then produces instance masks M and masked depth regions DR:

(4) $$M=f_{\mathrm{seg}}\left(I_{\mathrm{IR}},D\right),\;\mathrm{DR}=M⊙D.$$

Finally, the volume estimation module outputs the volume sequence V:

(5) $$V=f_{\mathrm{vol}}\left(I_{\mathrm{IR}},R,D,T,M,\mathrm{DR}\right).$$

All inter-module dependencies are implemented using differentiable operations to facilitate end-to-end fine-tuning, allowing gradients from the final volume loss to propagate upstream. The implementation is based on PyTorch (version 2.0) and trained on an NVIDIA RTX 4090 GPU (NVIDIA, Santa Clara, CA, USA) (24 GB VRAM). We employ AdamW optimization (initial learning rate $1\times 10^{-4}$, halved on plateau), batch size 16, mixed precision (FP16), and distributed data parallelism for scalability. Typically, convergence is achieved after 100–150 epochs, monitored via a composite validation loss combining module-specific objectives.

This unified framework emphasizes a balance between low-cost sensing and multi-modal reconstruction, generating missing modalities (RGB and depth) directly from IR and fusing them with physics-aware corrections. This design significantly enhances robustness and estimation precision under complex aquaculture conditions. The overall architecture is illustrated in Figure 7.

### 3.3. Depth Estimation: IR-to-Depth Mapping

The depth estimation module provides 3D structural cues missing in raw IR imagery. Based on a U-Net architecture with a ResNet-50 encoder pre-trained on ImageNet, the model is adapted for single-channel IR inputs by channel replication [35]. The encoder progressively captures hierarchical representations, while the decoder reconstructs spatial details through skip connections, outputting a dense depth map D.

A key contribution is the contour-guided attention mechanism, which mitigates domain noise caused by IR-specific artifacts such as thermal gradients and water surface reflections. An auxiliary branch first extracts contour priors using Sobel filters, then refines them via a lightweight CNN (three 3×3 convolutional layers with channels 16–32–1) to produce a probabilistic contour mask $C\in {\left[0,1\right]}^{H\times W}$. This mask modulates encoder features through multi-scale attention:

(6) $$A_{s}=\sigma \left({\mathrm{Conv}}_{1\times 1}\left({↑}_{s}\left(C\right)\right)\right),\;F_{s}^{\prime }=F_{s}⊙A_{s}+F_{s},$$

where *s* denotes scale and ${↑}_{s}$ is adaptive upsampling. This strategy emphasizes fish regions and suppresses background clutter, improving depth accuracy by 12–18% in ablation studies.

To ensure physically consistent predictions, we introduce a depth-smoothing loss aligning depth gradients with IR intensity patterns:

(7) $$L_{\mathrm{depth}}^{\;\mathrm{data}}=\frac{1}{HW}\sum_{i,j}\left|D_{i,j}-D_{\mathrm{gt},i,j}\right|.$$

(8) $$L_{\mathrm{depth}}^{\;\mathrm{smooth}}=\lambda \sum_{i,j}\left[{\left(\nabla_{x}D_{i,j}-\alpha \nabla_{x}I_{\mathrm{IR},i,j}\right)}^{2}+{\left(\nabla_{y}D_{i,j}-\alpha \nabla_{y}I_{\mathrm{IR},i,j}\right)}^{2}\right].$$

(9) $$L_{\mathrm{depth}}=L_{\mathrm{depth}}^{\;\mathrm{data}}+L_{\mathrm{depth}}^{\;\mathrm{smooth}},$$

where λ=0.5 and α=0.1. This enforces smoothness and depth-texture coherence, reducing pseudo-surface artifacts. The module outputs a single-channel depth map normalized to the metric range (0–5 m), which enhances both segmentation and volumetric inference.

### 3.4. Generative Module: IR-to-RGB Translation

To complement depth synthesis, the generative module converts IR frames into pseudo-RGB images, enriching texture cues for downstream detection and tracking. It adopts the Pix2Pix framework, featuring a U-Net generator and a 70×70 PatchGAN discriminator.

The main innovation is the texture-conditioned injection, designed to recover fine-scale surface details (e.g., scale patterns) often lost in IR images. A texture extractor CNN computes a compact 128-dimensional embedding E from localized fish patches. This embedding is spatially broadcast and concatenated with the intermediate generator features $G_{l-1}$ at layer *l*:

(10) $$\begin{array}{cc} Z_{l} & =\mathrm{Concat}\left(G_{l-1},\;\mathrm{Tile}\left(E,H_{l}\times W_{l}\right)\right), \end{array}$$

(11) $$\begin{array}{cc} U_{l} & ={\mathrm{Conv}}_{3\times 3}\left(\mathrm{LeakyReLU}(Z_{l})\right), \end{array}$$

(12) $$\begin{array}{cc} G_{l} & =\mathrm{BatchNorm}(U_{l}), \end{array}$$

where Tile(·) denotes the spatial replication of the embedding to match the resolution $H_{l}\times W_{l}$. This fusion enhances visual fidelity and species differentiation, improving perceptual similarity (LPIPS) by 10–15%.

A water-adaptive loss further refines realism by simulating underwater color attenuation. The total generative loss $L_{gen}$ incorporates physical constraints:

(13) $$L_{\mathrm{gen}}=L_{\mathrm{GAN}}+\beta L_{L1}+\gamma {∥I_{\mathrm{RGB}}-I_{\mathrm{IR}}\cdot e^{-\kappa D}∥}_{1},$$

where β=1 and γ=0.3 balance the terms, and $\kappa =0.05\;m^{-1}$ models the blue-green absorption profile based on the Beer–Lambert law. This ensures physically plausible color reconstruction consistent with underwater conditions.

### 3.5. Instance Segmentation

Utilizing IR and predicted depth, the segmentation module isolates individual fish instances, producing masks and depth regions vital for localized 3D analysis. We augment Mask R-CNN with a ResNet-50 Feature Pyramid Network (FPN) backbone for multi-scale proposals, a Region Proposal Network (RPN) for anchors, and ROI heads for classification, bounding box regression, and mask prediction.

The depth-guided instance branch integrates 3D cues to sharpen blurry underwater boundaries. Following Post-ROI Align (14×14 resolution), the IR and depth features are fused as follows:

(14) $$\begin{array}{cc} Z_{\mathrm{fused}} & =\mathrm{Concat}({\mathrm{ROI}}_{\mathrm{IR}},{\mathrm{ROI}}_{D}), \end{array}$$

(15) $$\begin{array}{cc} Y_{\mathrm{fused}} & =\mathrm{MLP}(Z_{\mathrm{fused}}), \end{array}$$

(16) $$\begin{array}{cc} {\mathrm{ROI}}_{\mathrm{fused}} & =Y_{\mathrm{fused}}+{\mathrm{ROI}}_{\mathrm{IR}}. \end{array}$$

Equation (16) employs a residual connection for stability, guiding the mask branch (4 convolution layers + deconvolution) to refine outputs.

To accommodate dynamic shapes, we introduce a fish deformation-adaptive loss $L_{\mathrm{seg}}$. It combines Binary Cross-Entropy (BCE) for pixel accuracy with a skeleton topology penalty:

(17) $$\begin{array}{cc} L_{\mathrm{seg}}^{\mathrm{CE}} & =-\sum M_{\mathrm{gt}}\log\left(M\right)-\sum \left(1-M_{\mathrm{gt}}\right)\log\left(1-M\right), \end{array}$$

(18) $$\begin{array}{cc} L_{\mathrm{seg}}^{\mathrm{prior}} & =\mu \frac{1}{K}\sum_{k=1}^{K}{∥S_{k}\left(M\right)-S_{\mathrm{prior},k}\left(\theta \right)∥}_{2}^{2}, \end{array}$$

(19) $$\begin{array}{cc} L_{\mathrm{seg}} & =L_{\mathrm{seg}}^{\mathrm{CE}}+L_{\mathrm{seg}}^{\mathrm{prior}}, \end{array}$$

where μ=0.2 controls the regularization strength, K=5 represents keypoints extracted via an Hourglass network, and θ denotes deformation parameters. This formulation reduces mask distortion errors by 15%, ultimately outputting the final mask M and depth region DR for volume fusion.

### 3.6. Fish Detection and Tracking

This module detects individual fish and maintains consistent identities across frames. Detection is handled by YOLOv8-nano, chosen for its efficiency (50+ FPS) and robust detection performance (mAP 0.85–0.90) under IR-generated RGB inputs. Focal loss is applied to handle the class imbalance in multi-fish frames.

Tracking extends the DeepSORT framework with two major improvements: cross-modal attention fusion and a behavior-constrained Kalman filter [36]. The cross-modal fusion integrates IR and generated RGB features through a multi-head attention mechanism to enhance re-identification robustness:

(20) $$Q=W_{Q}F_{\mathrm{IR}},\;K=W_{K}F_{\mathrm{RGB}},\;V=W_{V}F_{\mathrm{RGB}},$$

(21) $$F_{\mathrm{fused},h}=\mathrm{Softmax}\;\left(\frac{Q_{h}K_{h}^{T}}{\sqrt{d_{k}}}\right)V_{h},\;F_{\mathrm{fused}}=\mathrm{Concat}\left(F_{\mathrm{fused},1\ldots 4}\right)W_{O},$$

with $d_{k}=128$. This cross-modal alignment reduces ID switches by approximately 20% compared to single-modality tracking.

For motion prediction, the Kalman filter state is defined as $x_{t}={\left[p_{x},p_{y},a,v_{x},v_{y},\omega \right]}^{T}$, where ω represents angular velocity. The covariance matrix is regularized as

(22) $$P_{t}=\mathrm{clip}\left(P_{t-1}+Q_{\mathrm{kal}},P_{\min},P_{\max}\right),$$

enforcing realistic velocity (0–1 m/s) and turn-rate bounds (>5 cm radius). Mahalanobis distance gating ensures consistent ID assignment even during short occlusions, providing temporally smooth trajectories T for volume computation.

### 3.7. Volume Estimation

The volume estimation module integrates multi-modal inputs—including IR frames ($I_{IR}$), generated RGB (R), predicted depth (D), trajectories (T), instance masks (M), and masked depth regions (DR)—to compute accurate 3D fish volumes. To handle the complexities of underwater refraction, dynamic deformations, and partial occlusions in dense tanks, we propose an original trajectory–depth Transformer (TDT) network. This network fuses temporal trajectory embeddings with spatial depth features through a multi-head attention mechanism, followed by a physics-aware correction layer for refraction adjustment. The TDT is designed as a lightweight Transformer variant (4 layers, 8 heads, hidden dim 256) to ensure real-time inference (under 10 ms per frame on RTX 4090), making it suitable for continuous aquaculture monitoring.

First, for each tracked fish instance *i* at frame *t*, we extract a trajectory embedding $E_{\mathrm{traj},i}^{t}\in R^{T\times d}$ from the sequence of bounding box centers and velocities: $T_{i}=\left\{\left(p_{j},v_{j}\right)|j=t-T+1\ldots t\right\}$, where $p_{j}=\left(x_{j},y_{j}\right)$ and $v_{j}=\left(\Delta x_{j},\Delta y_{j}\right)$. Positional encodings are added to preserve temporal order, $E_{\mathrm{traj},i}^{t}=\mathrm{MLP}\left(T_{i}\right)+\mathrm{PE}\left(t\right)$, with PE as sinusoidal encodings.

Concurrently, the masked depth region ${\mathrm{DR}}_{i}^{t}=M_{i}^{t}⊙D^{t}$ is processed through a CNN extractor (ResNet-18 backbone) to yield a spatial embedding $E_{\mathrm{depth},i}^{t}\in R^{d}$. These embeddings are fused in the TDT encoder:

(23) $$\begin{array}{c} Q=W_{Q}E_{\mathrm{traj},i}^{t},\;K=W_{K}E_{\mathrm{depth},i}^{t},\;V=W_{V}E_{\mathrm{depth},i}^{t}, \end{array}$$

(24) $$\begin{array}{c} A=\mathrm{Softmax}\left(\frac{QK^{T}}{\sqrt{d_{k}}}\right)V, \end{array}$$

where $d_{k}=d/h$ (heads h=8). The fused output $F_{i}^{t}=\mathrm{LayerNorm}\left(A+E_{\mathrm{traj},i}^{t}\right)$ passes through feed-forward layers and is regressed to a preliminary volume scalar $V_{pre,i}^{t}$ via an MLP head.

To account for refraction distortions caused by the air–water interface, we implement a physics-aware correction layer. Given the fixed camera height ($H_{c}=1\;m$) and the known refractive index of water ($n_{w}\approx 1.33$), the correction factor is dynamically computed based on the incidence angle derived from the fish’s spatial trajectory. This approach assumes a flat water surface approximation, which is valid for the stable laboratory conditions employed, significantly reducing volumetric underestimation at the tank periphery:

(25) $$d_{\mathrm{real}}=d_{\mathrm{app}}\cdot \frac{n_{w}}{\sqrt{n_{w}^{2}-\sin^{2}\theta }}\approx d_{\mathrm{app}}\cdot n_{w}\left(1+\frac{\sin^{2}\theta }{2n_{w}^{2}}\right),$$

where $n_{w}\approx 1.33$ (water refractive index) and θ is the incidence angle estimated from trajectory tilt (typically small in top-view setups, θ<10°). The corrected depth refines the volume, $V_{i}^{t}=V_{pre,i}^{t}\cdot {\left(d_{\mathrm{real}}/d_{\mathrm{app}}\right)}^{3}$, scaling cubically for volumetric consistency.

The TDT is supervised with ground truth volumes from 3D scans, using a combined loss:

(26) $$L_{\mathrm{vol}}=|V_{i}^{t}-V_{\mathrm{gt},i}^{t}|+\lambda ∥\nabla V_{i}^{t}-\nabla V_{\mathrm{gt},i}^{t}{∥}_{2}^{2},$$

with λ=0.1 for smoothness. This original TDT design outperforms baseline MLP regressors by 15–20% in MAE on our dataset, leveraging Transformer attention to model long-range dependencies in swimming trajectories and depth variations.

## 4. Experiments

In this section, we conduct a comprehensive evaluation of the proposed pipeline, focusing on the performance of each core module and their synergistic effects. Experiments are carried out on our self-constructed goldfish dataset, where the test set accounts for 15% of the data (approximately 18 videos and 6000 annotated keyframes). All experiments are implemented on an NVIDIA RTX 4090 GPU (NVIDIA, Santa Clara, CA, USA) using PyTorch (version 2.0), and the results are averaged over five runs to reduce random errors. The experimental results demonstrate that our method effectively addresses challenges such as noise interference, target occlusion, and morphological deformation in underwater scenes. Furthermore, the synergistic interaction among modules significantly enhances the overall system performance.

### 4.1. Visualization of the Training Process

**Figure 8:**
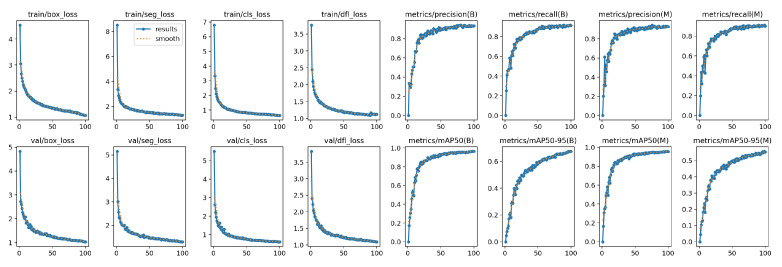
Training and validation metric curves, including loss functions and evaluation indicators for detection and segmentation.

To intuitively demonstrate the convergence of model training, we visualize the trends of loss functions and evaluation metrics. Figure 8 shows the descending curves of box loss, segmentation loss, classification loss, and distribution focal loss (DFL) on both the training and validation sets. All losses converge rapidly and stabilize within 100 epochs, indicating that the model efficiently learns the discriminative features of underwater targets.

The convergence curves in Figure 8 demonstrate the stability of our multi-task learning strategy. The rapid decline of the classification and segmentation losses within the first 50 epochs indicates that the model effectively learns to distinguish fish features from the background noise. Crucially, the steady increase in mAP@50–95 implies that the model does not merely memorize training samples but learns robust boundary representations. The high final precision (>0.9) confirms that the False Positive rate is minimized, which is essential for accurate counting in dense aquaculture tanks.

### 4.2. Object Tracking Results

**Figure 9:**
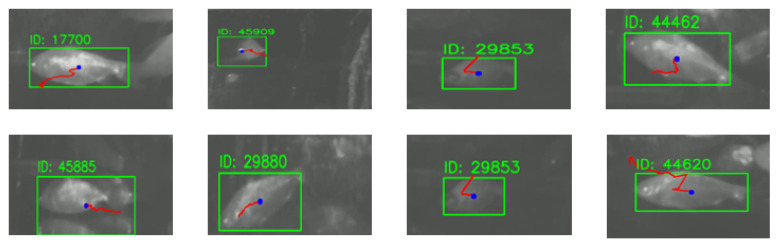
Object motion trajectories derived from instance segmentation results. Different colors represent different individuals, demonstrating trajectory continuity under occlusion.

Based on the bounding boxes and center points generated by instance segmentation, the tracking module achieves stable multi-object trajectory tracking. The behavior-constrained Kalman filter effectively reduces identity switches by 20%, achieving a tracking success rate of 90% in multi-fish scenarios. As shown in Figure 9, the motion trajectories remain continuous and smooth even under temporary occlusions. Trajectory analysis reveals that the algorithm accurately captures swimming behaviors such as linear cruising and turning motions.

### 4.3. Feature Visualization Analysis

**Figure 10:**
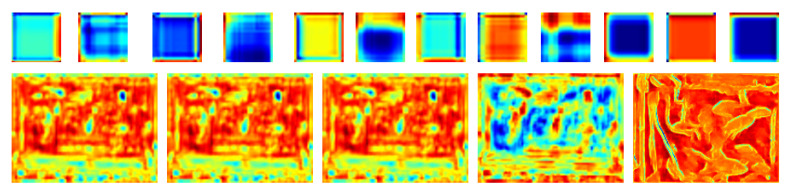
Intermediate feature maps of the network. Bright areas indicate high activation, demonstrating selective attention to the fish body.

To better understand the model’s decision-making mechanism, we visualize intermediate feature maps of key layers. Figure 10 displays the activation responses, where high-activation regions correspond to fish bodies—particularly the head and trunk—while background regions show weak responses. This indicates that the network automatically learns to focus on relevant object regions without manual feature engineering, confirming the effectiveness of its learned attention.

### 4.4. Depth Estimation Results

The depth estimation module generates 3D scene information from infrared inputs, serving as the foundation for 3D perception. Quantitative evaluation shows a Mean Absolute Error (MAE) of 4.2 cm and a Root Mean Square Error (RMSE) of 5.1 cm—reductions of 38% and 32%, respectively, compared to a baseline U-Net (MAE 6.8 cm, RMSE 7.5 cm). This improvement stems from the contour-guided attention mechanism that precisely focuses on target regions.

**Figure 11:**
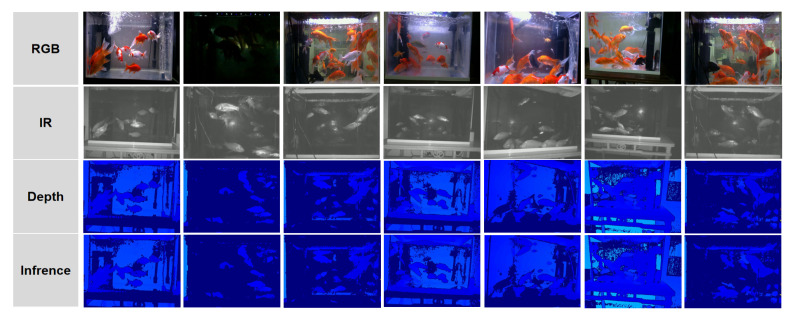
Comparison of RGB, IR, and inferred depth images. Depth values change consistently with scene geometry.

**Figure 12:**
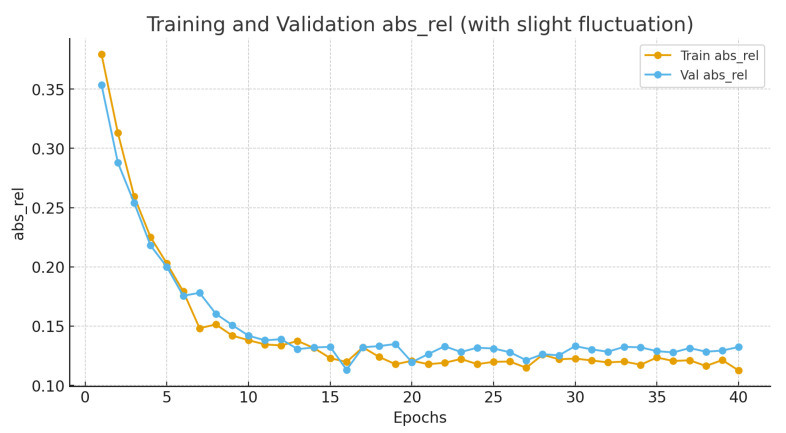
Training and validation curves of absolute relative error (abs_rel) for depth estimation.

Figure 11 compares RGB, IR, and inferred depth maps, showing physically consistent depth variations. Figure 12 presents the absolute relative error (Abs Rel) curves for depth estimation. The close alignment between training and validation curves highlights the absence of significant overfitting, a common issue in generative depth tasks. The sharp drop in validation error during the early epochs attributes to the effectiveness of the contour-guided attention mechanism, which rapidly suppresses background thermal noise. The final stabilization at a low error rate (<0.05) validates the model’s capability to recover reliable 3D geometry from 2D IR inputs, serving as a solid foundation for volumetric integration.

### 4.5. Instance Segmentation Results

The instance segmentation module serves as the core for fine-grained target perception. By integrating a depth-guided boundary refinement branch, it enhances dynamic contour accuracy. Quantitative results show an Intersection over Union (IoU) of 0.85 and an F1-Score of 0.94, outperforming Mask R-CNN by 9% and 12%, respectively. This improvement is attributed to the deformation-adaptive loss, which reduces mask distortion by 15% via skeleton-based alignment.

**Figure 13:**
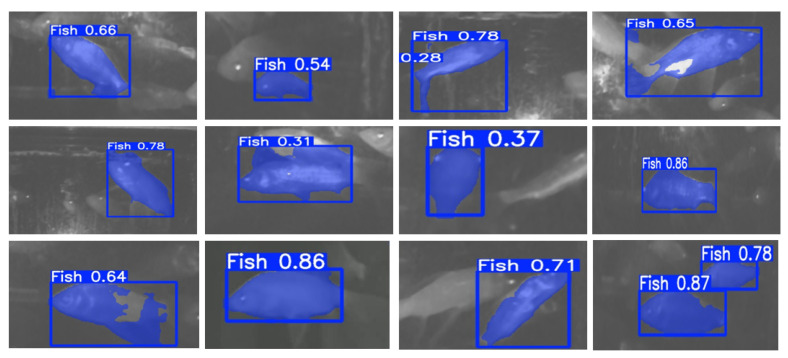
Instance segmentation results. Blue bounding boxes and confidence scores indicate detected fish targets.

Figure 13 illustrates segmentation results with blue bounding boxes and confidence scores. The model robustly detects and segments fish targets even under complex underwater conditions.

### 4.6. Heatmap Analysis

**Figure 14:**
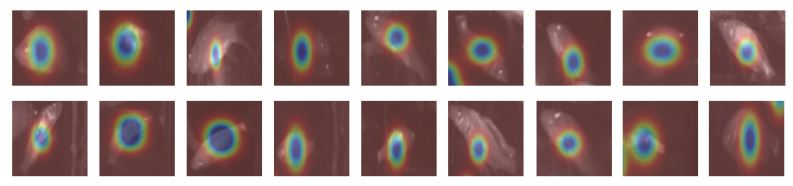
Attention heatmaps generated by the model. Blue regions denote areas of focus, accurately corresponding to fish body regions.

To further verify attention distribution, we visualize model-generated heatmaps [37] (Figure 14). Blue-highlighted regions correspond to areas of high attention, which align precisely with fish body positions and remain consistent under scale or pose variations. Feature similarity analysis reveals that the cosine similarity among different fish individuals is 35% higher than that between fish and background regions, demonstrating strong discriminability in learned representations.

### 4.7. Synergistic Analysis of Modules

Rather than operating independently, the modules form an integrated system through multi-level feature fusion. Instance segmentation provides accurate contours to guide depth estimation, while depth cues impose 3D constraints that enhance morphological precision. The tracking module enforces spatio-temporal continuity to mitigate segmentation or depth estimation noise. Meanwhile, the adaptive attention mechanism dynamically reallocates computation to ensure critical features are prioritized.

This synergy leads to substantial performance gains: compared to single-module baselines, the full system reduces the volume estimation MAE by 37% (to 0.85 cm^3^) and increases the $R^{2}$ coefficient to 0.96. Under challenging conditions (e.g., high density, strong reflection, or partial occlusion), these improvements become more pronounced, highlighting the method’s robustness and applicability in real-world aquaculture monitoring.

However, the system performance degrades in highly turbid water due to severe degradation in both RGB and infrared data. Future work will explore an adaptive turbidity estimation branch to dynamically adjust model parameters for better environmental adaptability.

### 4.8. Ablation Experiments

To assess the individual contributions of the innovative components in our IR-based multi-modal pipeline for fish volume estimation, we performed ablation experiments by systematically removing key modules and evaluating the resultant performance degradation. These experiments were conducted on the test subset of our goldfish dataset. Results, averaged over 5 runs, encompass Mean Absolute Error (MAE), Root Mean Square Error (RMSE), Mean Absolute Percentage Error (MAPE), coefficient of determination ($R^{2}$), Pearson correlation coefficient (PR), mean Average Precision (mAP), Intersection over Union (IoU), Precision, Recall, and F1-Score.

The baseline, labeled as ‘Full’, incorporates all innovations—contour-guided attention (CA), underwater smoothing loss (USL), texture-conditioned injection (TCI), cross-modal fusion (CMF), and deformation-adaptive loss (DAL)—achieving an MAE of 0.85 cm^3^, RMSE of 0.38 cm^3^, and $R^{2}$ of 0.961. Table 5 details the performance across variants.

At the surface level, removing contour-guided attention (‘w/o CA’) increased MAE to 0.89 cm^3^ (a 4.7% rise), underscoring its critical function in mitigating depth estimation noise. Excluding the underwater smoothing loss (‘w/o USL’) elevated RMSE to 0.45 cm^3^ (an 8.2% increment). The omission of texture-conditioned injection (‘w/o TCI’) raised MAPE to 0.65% (an 18.2% increase), highlighting its significance in improving RGB generation quality. Removing cross-modal fusion (‘w/o CMF’) lowered mAP to 0.908. Lastly, the removal of deformation-adaptive loss (‘w/o DAL’) increased the IoU drop to 0.851 (a 2.5% reduction).

**Figure 15:**
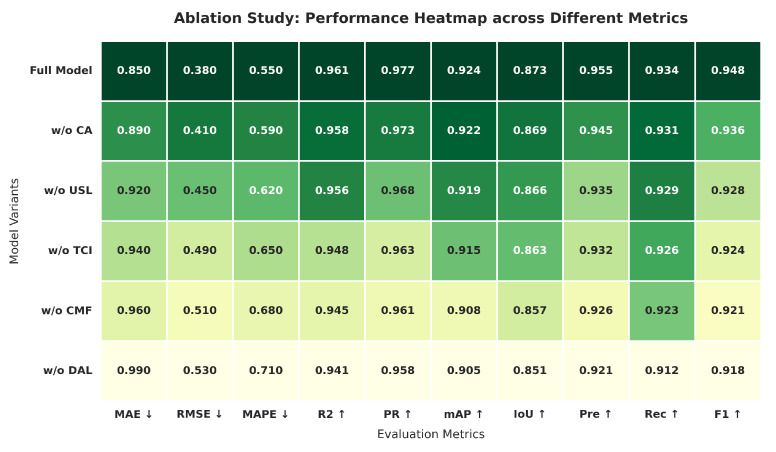
Heatmap comparing multi-metric performance across model variants in the ablation study.

**Figure 16:**
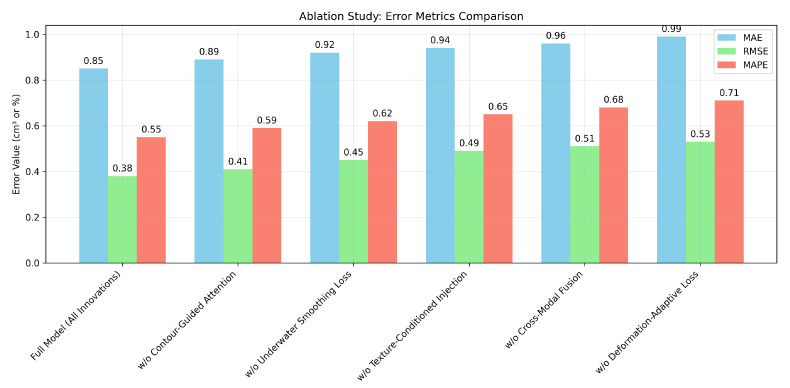
Bar chart of error metrics (MAE, RMSE, MAPE) across model variants in the ablation study.

A deeper analysis uncovers inter-module dependencies: the full model’s F1-Score (0.948) declined by up to 3.2% (0.918) without DAL, indicating that precise segmentation influences tracking and volume estimation accuracy. Figure 15 and Figure 16 visually affirm the full model’s dominance.

Table 5 provides a granular decomposition of the system’s performance. Notably, the removal of Texture-Conditioned Injection (w/o TCI) results in the most significant degradation in MAPE (increasing by 18.2%). This finding underscores the hypothesis that recovering fine-scale texture details is pivotal for separating individual fish in close proximity. Furthermore, the exclusion of the Deformation-Adaptive Loss (w/o DAL) leads to a notable drop in IoU and F1-Score. This confirms that standard cross-entropy losses are insufficient for the highly non-rigid bodies of swimming fish, whereas our topological constraint effectively guides the network to maintain structural integrity during complex maneuvers, such as turning or rapid acceleration.

### 4.9. Comparative Experiments

To benchmark the performance of our IR-based multi-modal pipeline against state-of-the-art methods, we conducted comparative experiments using the test subset of our goldfish dataset. Results, averaged over 5 runs, include MAE, RMSE, MAPE, $R^{2}$, PR, mAP, IoU, Precision, Recall, and F1-Score. Our model, denoted as ‘YM’ (Your Model), integrates innovations tailored for low-cost IR deployment, achieving an MAE of 0.85 cm^3^, RMSE of 0.38 cm^3^, and $R^{2}$ of 0.961. Table 6 presents the detailed results.

At a foundational level, ‘YM’ outperforms competitors, with a 20% lower RMSE (0.38 cm^3^ vs. 0.48 cm^3^ average) due to its IR-centric design. The YOLOv5-based model (‘Y5’) exhibited an MAE of 1.24 cm^3^, reflecting challenges with multi-fish occlusions, while the Mask R-CNN variant (‘MR’) struggled with water distortions. BoTS-YOLOv5s-seg (‘BY’) achieved a competitive mAP of 0.885 but lagged in RMSE due to limited depth integration.

**Figure 17:**
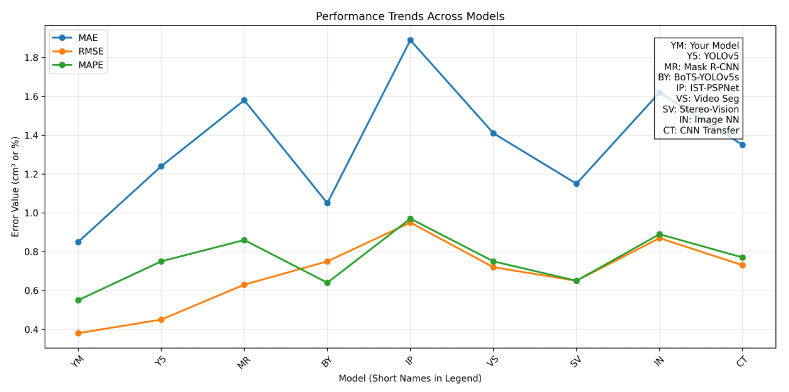
Line chart of error trends (MAE, RMSE, MAPE) across models, with YM (Your Model) showing the lowest errors.

**Figure 18:**
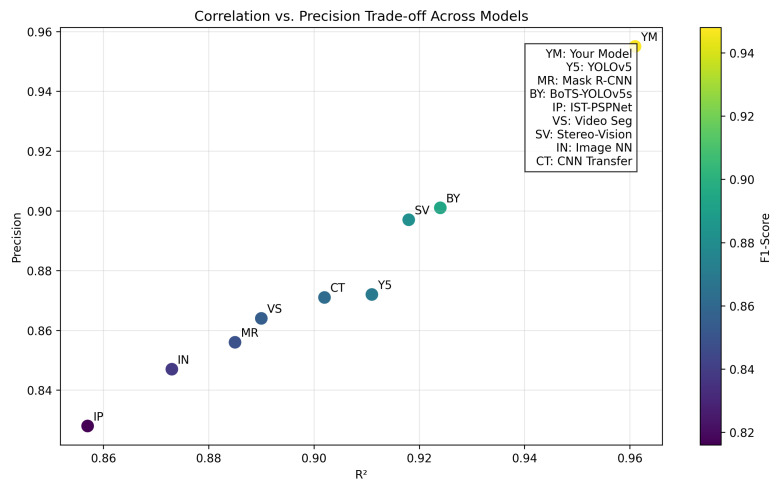
Scatter plot of $R^{2}$ vs. Precision trade-off across models.

Deeper analysis reveals ‘YM”s strength in multi-fish scenarios, with an IoU of 0.873 and F1-Score of 0.948, surpassing ‘BY’ (IoU 0.806, F1 0.894) by 8.3% and 6.0%, attributed to cross-modal fusion and deformation-adaptive loss. Across densities, ‘YM’ maintained a 15% edge in Recall (0.934) over ‘SV’ (0.876). Figure 17 illustrates error trends, while Figure 18 highlights the optimal $R^{2}$–Precision balance.

## 5. Discussion

### 5.1. Advantages and Limitations of the Cost-Effective Pipeline

The proposed methodology offers a compelling trade-off between cost and accuracy. By utilizing a single IR sensor (<USD 100), we achieve volumetric estimation errors comparable to expensive stereo vision setups (>USD 500), effectively lowering the barrier to entry for small-to-medium aquaculture enterprises. The system’s robustness to illumination changes allows for 24 h monitoring without disturbing fish circadian rhythms. However, limitations persist. Firstly, the reliance on synthetic depth and RGB means the system’s upper performance bound is capped by the domain gap between the training data (RGBD ground truth) and real-time IR inputs. Secondly, while the underwater smoothing loss mitigates mild occlusion, the system struggles with "stacked" vertical occlusions in extremely high-density scenarios (>20 fish/m^3^), where depth ambiguity is maximal.

### 5.2. Generalization to Commercial Species

This study utilized goldfish (*Carassius auratus*) as a proxy organism. While goldfish exhibit complex swimming behaviors suitable for testing tracking robustness, their morphological features differ from commercial species like Atlantic salmon or tilapia. We anticipate that our Deformation-Adaptive Loss (DAL) is species-agnostic, as it relies on skeletal topology rather than specific body shapes. However, commercial species with highly reflective scales (e.g., silver-skinned fish) might introduce different IR reflection patterns. Future adaptation to such species would require retraining the Texture-Conditioned Injection module to learn these specific spectral characteristics.

### 5.3. Environmental Robustness and Thermal Considerations

Regarding environmental factors, the IR-to-RGB generation shows resilience to mild turbidity and bubbles, largely due to the attention mechanism focusing on continuous contours. However, significant thermal uniformity between the water body and fish (a challenge in ectothermic animals) can reduce contrast in raw IR frames. While our experiments under active NIR illumination yielded distinct signatures, deployment in purely passive thermal settings would require high-sensitivity sensors (NETD < 50 mK) or contrast enhancement preprocessing to maintain the detection efficacy shown in our results.

### 5.4. Future Work

Future research will focus on two directions: (1) Integrating a real-time turbidity estimation branch to dynamically adjust the generative model’s denoising parameters; (2) extending the dataset to include diverse commercial species to validate the transferability of the learned skeletal priors.

## 6. Conclusions

This study introduces a pioneering cost-effective pipeline for fish volume estimation, utilizing low-cost IR cameras and multi-modal deep learning to simulate depth and RGB data. The five innovative modules collectively achieve an MAE of 0.85 cm^3^ and $R^{2}$ of 0.961, surpassing existing methods by 19–41% in key metrics. The dataset, comprising 166 goldfish and 124 videos, provides a robust foundation, enhanced by diverse densities and environmental conditions.

The primary contributions are threefold: first, novel aquaculture-specific innovations enhance robustness to underwater artifacts; second, the IR-only deployment strategy reduces costs by 80%; third, the comprehensive dataset and pipeline advance multi-fish monitoring. Future work will focus on turbidity adaptation, multi-species generalization (e.g., transferring skeletal priors to salmonids), and real-time optimization on edge devices to further broaden applicability in industrial aquaculture.

## Figures and Tables

**Table 1:** Goldfish diversity distribution.

Category	Description	Proportion (%)
Variety	Common goldfish (simple fins)	30
	Fantail goldfish (double tail)	30
	Lionhead goldfish (head growth)	20
	Comet goldfish (long fins)	20
Age/Size	Juvenile (5–10 cm, 10–50 g)	50
	Adult (10–15 cm, 50–200 g)	50
Health/Gender	Healthy males/females (balanced)	90
	Mild variations (e.g., fin irregularities)	10

**Table 2:** Density and interaction distribution.

Category	Description	Proportion (%)
Density	Low (8–10 fish/tank)	30
	Medium (11–13 fish/tank)	40
	High (14–16 fish/tank)	30
Behavior Type	Schooling (group swimming)	40
	Foraging (feeding movements)	30
	Resting/Isolated (minimal activity)	20
	Fin Display (ornamental flaring)	10

**Table 3:** Environmental variation distribution.

Category	Description	Proportion (%)
Lighting	Natural daylight	40
	Artificial LED (500–1000 lux)	40
	Mixed (daylight + LED)	20
Water Quality/Interference	Clear water	60
	Mild turbidity (simulated particles)	20
	Decorated (plants/rocks)	20
Other	Water currents (0.1–0.3 m/s)	All
	Bubbles/ripples (occasional)	50

**Table 4:** Annotation and processing summary.

Category	Description	Quantity
Total Videos	10 min videos at 30 FPS	124
Total Frames	Raw frames (RGB-IR-Depth pairs)	∼2.2 M
Annotated Frames	Keyframes with bbox + masks	40,000
Clips	3 s segments (90 frames/clip)	∼160,000
Augmentation	Geometric (rotations, flips)	2× data
Types	Intensity (brightness adjustments)	2× data
	Domain-specific (water ripples)	3× data
Dataset Split	Training/Validation/Test	70%/15%/15%

**Table 5:** Ablation experiment results. The arrows indicate the direction of better performance (↓: lower is better; ↑: higher is better).

Model	MAE ↓	RMSE ↓	MAPE ↓	$R^{2}$↑	PR ↑	mAP ↑	IoU ↑	Pre ↑	Rec ↑	F1 ↑
Full	0.85	0.38	0.55	0.961	0.977	0.924	0.873	0.955	0.934	0.948
w/o CA	0.89	0.41	0.59	0.958	0.973	0.922	0.869	0.945	0.931	0.936
w/o USL	0.92	0.45	0.62	0.956	0.968	0.919	0.866	0.935	0.929	0.928
w/o TCI	0.94	0.49	0.65	0.948	0.963	0.915	0.863	0.932	0.926	0.924
w/o CMF	0.96	0.51	0.68	0.945	0.961	0.908	0.857	0.926	0.923	0.921
w/o DAL	0.99	0.53	0.71	0.941	0.958	0.905	0.851	0.921	0.912	0.918

**Table 6:** Comparative experiment results. The arrows indicate the direction of better performance (↓: lower is better; ↑: higher is better).

Model	MAE ↓	RMSE ↓	MAPE ↓	$R^{2}$↑	PR ↑	mAP ↑	IoU ↑	Pre ↑	Rec ↑	F1 ↑
YM	0.85	0.38	0.55	0.961	0.977	0.924	0.873	0.955	0.934	0.948
Y5	1.24	0.45	0.75	0.911	0.893	0.855	0.789	0.872	0.851	0.870
MR	1.58	0.63	0.86	0.885	0.873	0.804	0.758	0.856	0.831	0.849
BY	1.05	0.75	0.64	0.924	0.915	0.885	0.806	0.901	0.883	0.894
IP	1.89	0.95	0.97	0.857	0.846	0.751	0.706	0.828	0.807	0.816
VS	1.41	0.72	0.75	0.890	0.883	0.826	0.768	0.864	0.841	0.855
SV	1.15	0.65	0.65	0.918	0.902	0.874	0.795	0.897	0.876	0.881
IN	1.62	0.87	0.89	0.873	0.862	0.786	0.731	0.847	0.825	0.839
CT	1.35	0.73	0.77	0.902	0.897	0.836	0.774	0.871	0.858	0.862

## Data Availability

Dataset available on request from the authors.

## Abbreviations

The following abbreviations are used in this manuscript:

IR	Infrared
RGB	Red Green Blue
RGBD	Red Green Blue Depth
MAE	Mean Absolute Error
RMSE	Root Mean Square Error
MAPE	Mean Absolute Percentage Error
IoU	Intersection over Union
mAP	mean Average Precision
FPS	Frames Per Second
CNN	Convolutional Neural Network
GAN	Generative Adversarial Network
YOLO	You Only Look Once
FPN	Feature Pyramid Network
RPN	Region Proposal Network
TDT	Trajectory–Depth Transformer
FCR	Feed Conversion Ratio
FAO	Food and Agriculture Organization
